# Influence of EEG Signal Augmentation Methods on Classification Accuracy of Motor Imagery Events

**DOI:** 10.3390/s26041258

**Published:** 2026-02-14

**Authors:** Bartłomiej Sztyler, Aleksandra Królak, Paweł Strumiłło

**Affiliations:** Institute of Electronics, Lodz University of Technology, 90-924 Lodz, Poland; bartlomiej.sztyler@p.lodz.pl (B.S.)

**Keywords:** motor imagery, EEG decoding, deep learning, CNN, data augmentation

## Abstract

This study investigates the impact of various data-augmentation techniques on the performance of neural networks in EEG-based motor imagery three-class event classification. EEG data were obtained from a publicly available open-source database, and a subset of 25 patients was selected for analysis. The classification task focused on detecting two types of motor events: imagined movements of the left hand and imagined movements of the right hand. EEGNet, a convolutional neural network architecture optimized for EEG signal processing, was employed for classification. A comprehensive set of augmentation techniques was evaluated, including five time-domain transformations, three frequency-domain transformations, two spatial-domain transformations and two generative approaches. Each method was tested individually, as well as in selected two- and three-method cascade combinations. The augmentation strategies were tested using three data-splitting methodologies and applying four ratios of original-to-generated data: 1:0.25, 1:0.5, 1:0.75 and 1:1. Our results demonstrate that the augmentation strategies we used significantly influence classification accuracy, particularly when used in combination. These findings underscore the importance of selecting appropriate augmentation techniques to enhance generalization in EEG-based brain–computer interface applications.

## 1. Introduction

Motor execution (ME) and motor imagery (MI) are fundamental concepts in neuroscience, referring to the processes of actually performing a movement and mentally rehearsing a movement without physical execution, respectively. Both processes are accompanied by neural activity patterns, detectable in the sensorimotor cortex of the brain [1]. The ability to detect and decode these patterns is important particularly in the field of neurorehabilitation [2], but also for patients recovering from strokes [3] or other neurological injuries. Using the decoding of MI and ME signals, brain–computer interfaces (BCIs) can provide means for individuals with severe motor impairments to interact with their environment, facilitating functional recovery and improving quality of life.

A variety of techniques are available to measure brain activity, including fMRI (functional Magnetic Resonance Imaging) [4], MEG (magnetoencephalography) [5], and EEG (electroencephalography) [6]. Although fMRI and MEG offer very good spatial resolution, they are often expensive, require specialized infrastructure, and are not portable. In contrast, EEG is a non-invasive, cost-effective, and portable method with very good temporal resolution. It records the electrical potentials generated by the brain, allowing for real-time brain activity analysis.

The decoding of MI and ME signals has been a subject of extensive research, with various methodologies developed over the years [7,8,9,10,11,12,13]. Early approaches relied on traditional signal-processing techniques [8,14,15,16] and machine learning algorithms [17] like Support Vector Machines (SVMs) [18], Independent Component Analysis [19] or Linear Discriminant Analysis (LDA) [20,21]. However, the rapid development of deep learning has revolutionized this field. Neural network architecture, such as convolutional neural networks (CNNs) [22], has demonstrated very good performance in capturing the spatio-temporal features of EEG signals, leading to state-of-the-art results in MI and ME classification [23,24,25,26].

The complexity of a BCI’s decoding task grows with the number of classes it must distinguish. Many studies limit themselves to binary distinctions, such as left-hand versus right-hand movement or imagery, but real clinical systems require richer state discrimination [27]. In practice, hand and foot movements for both motor execution (ME) and motor imagery (MI) are often considered [28], yielding four primary classes (ME Left Hand, ME Right Hand, MI Left Hand, MI Right Hand) plus a Rest class that denotes no intended movement. As the class count increases, the decoding accuracy typically declines [29,30,31].

Our work focuses on a more realistic neurorehabilitation scenario: a three-class problem comprising Right Hand, Left Hand, and Rest [32]. This formulation mirrors the decision logic of practical BCIs, where the goal is to detect voluntary intent for either hand while suppressing false positives during rest periods. By evaluating data-augmentation strategies within this problem statement, we aimed to provide results that would be directly applicable to real-world clinical deployments.

A major challenge in training deep learning models is lack of high-quality, labeled EEG data [33,34,35]. This limitation often leads to overfitting, where models perform well on training data but fail to generalize to new, unseen data. Data augmentation, a technique used to artificially expand the training dataset, is a crucial strategy to overcome this issue. It involves generating new, synthetic data samples by applying various transformations to the existing data [36]. While data augmentation is a well-established practice in computer vision [37], its application in EEG analysis, particularly for MI and ME tasks, has recently gained popularity, and more and more publications are focused on researching its potential [38,39,40,41].

Despite widespread use of data augmentation, a significant gap remains in the literature. While some studies, such as the comparative analyses by [42,43,44], have systematically evaluated the effectiveness of various augmentation techniques, they often focus on single methods or a limited range of classification tasks. To date, there has been a lack of comprehensive studies that not only test a broad spectrum of augmentation techniques under a consistent experimental protocol but also investigate how these methods behave with different numbers of classes. The effects of combining multiple augmentation methods (e.g., cascade augmentation), the quantity of added artificial data, and the influence of different data-splitting strategies on final classification accuracy have not been thoroughly investigated. The lack of an extended analysis makes it difficult for researchers to select optimal augmentation strategies for specific tasks.

The aim of this paper was to fill this gap by presenting a comprehensive analysis of the impact of data augmentation on the accuracy of EEG-based MI classification. For our study, we systematically evaluated the performance of a deep learning model using a wide range of transformative and generative augmentation techniques. We analyzed not only the individual performance of these methods, but also their collective impact when applied in various cascading configurations. Furthermore, we present a detailed analysis of the impact of the amount of the augmented data on the classification results.

## 2. Materials and Methods

### 2.1. EEG Dataset

This study utilized the EEG Motor Movement/Imagery Database [45], a publicly available resource hosted on PhysioNet (PhysioNet EEG Motor Movement/Imagery Database) [46]. The dataset consists of over 1500 one- and two-minute electroencephalogram (EEG) recordings acquired from 109 healthy volunteers. Selection of this database was made upon its established recognition within the research community, its comprehensive documentation, and its frequent application in prior investigations evaluating motor imagery-based brain–computer interfaces (BCIs). Consequently, it served as a suitable benchmark dataset for assessing and comparing methodological approaches in this domain. Our data acquisition employed a 64-channel EEG system (BCI2000) [45], with a 10–10 system for electrode placement [47,48]. The raw EEG signals were provided in EDF+ format, sampled at fs = 160 Hz. Each subject participated in multiple experimental runs involving different types of motor activity, including: actual movements (e.g., opening and closing of the left or right fist); motor imagery (MI) tasks (e.g., imagining the same movements without physical execution).

Each subject participated in an experimental protocol comprising 14 runs: two one-minute baseline recordings (eyes open and eyes closed) followed by three two-minute trials for each of four motor tasks. These tasks encompassed both executed and imagined movements, specifically left-fist opening/closing, right-fist opening/closing, simultaneous fist closure, and simultaneous foot movement. Task initiation was cued visually via on-screen prompts indicating the target location. For this research, a subset of 25 subjects was randomly selected from the full database, which included 2250 epochs for analysis.

### 2.2. Data Preparation and Augmentation

In this research, our experiments were conducted by training the chosen deep learning model on both raw EEG data and data subjected to various augmentation techniques.

EEG preprocessing was performed in a minimal and transparent manner to preserve the original signal characteristics and to evaluate the robustness of the proposed approach on near-raw data. The preprocessing pipeline consisted of the following steps:

- Channel Configuration and Electrode Layout—EEG signals were recorded using 64 electrodes placed according to the international 10–10 system. The following electrodes were excluded from the analysis: Nz, F9, F10, FT9, FT10, A1, A2, TP9, TP10, P9, and P10. After exclusion, the remaining channels were used consistently across all subjects and experiments.
- Referencing—the EEG signals were analyzed using the original reference configuration provided by the acquisition system. This decision was motivated by the desire to avoid introducing additional spatial transformations and to assess the performance of the EEGNet architecture on minimally processed data.
- Filtering—two signal variants were analyzed: (i) raw EEG signals without any frequency-domain preprocessing, and (ii) EEG signals after applying a notch filter at 60 Hz to remove power line interference. No additional filtering was used, allowing the convolutional layers of the EEGNet to learn frequency-selective representations directly from the data.
- Artifact Handling—no explicit artifact rejection or correction procedures were applied. All recorded epochs were retained for analysis to reflect realistic acquisition conditions and to evaluate the model’s robustness against common EEG artifacts.
- Normalization—the EEGNet architecture internally incorporated batch normalization layers, which provided adaptive normalization during the training and reduced sensitivity to Amplitude Scaling across the subjects and channels.
- Class Balance Strategy and Dataset Splits—the dataset consisted of EEG recordings from 25 subjects performing three motor imagery tasks: left-hand movement imagination, right-hand movement imagination and rest state (no action). For each subject, 7–8 epochs were recorded for left-hand movement imagination, 7–8 epochs for right-hand movement imagination and 15 epochs for the relax state. This resulted in a total of 200 epochs for each class. To address class imbalance, the rest class was balanced to match the size of the left- and right-hand movement classes. A subset of rest state epochs was selected from the available pool, resulting in an equal number of samples for all three classes.

All the applied augmentation options were tested on both raw and notch-filtered data to comprehensively evaluate their combined impact on model performance. As a reference learning baseline, experiments were also conducted exclusively on clean, raw data, without any augmentation or preliminary filtering, to establish a comparative benchmark.

Minimal preprocessing was chosen to preserve the original signal characteristics and evaluate the robustness of EEGNet on near-raw data. This approach aligns with prior work in motor imagery EEG classification, where end-to-end CNN models trained on raw EEG achieved competitive performance and were able to implicitly learn discriminative features without extensive preprocessing [49,50]. By analyzing both raw and notch-filtered signals, we assessed the trade-off between signal-to-noise ratio improvement and retention of physiological information, while maintaining comparability with the established MI EEG literature.

To evaluate the proposed methods, three distinct data-splitting methodologies were tested, as detailed in Section 2.2.1. Additionally, a comprehensive set of twelve data-augmentation methods were investigated, comprising ten transformative techniques and two generative techniques. These augmentation methods were applied under three different strategies to explore their combined effects. Moreover, the study systematically examined the impact of the quantity of the augmented data on the overall classification performance, providing insights into the optimal balance between original and synthetic data.

#### 2.2.1. Data Splitting Methodology

**Figure 1:**
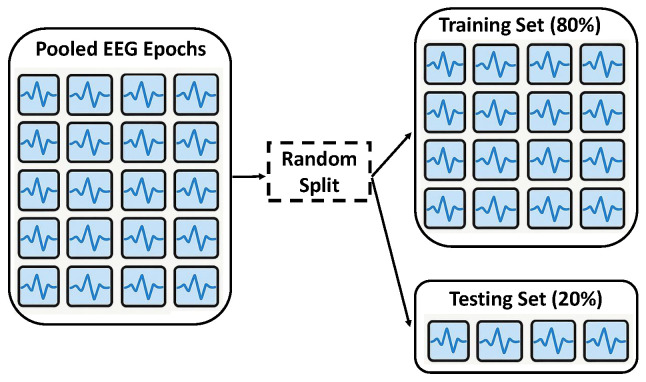
Illustration of Pooled Random Split methodology.

To ensure robust model generalization and prevent any data leakage between the training and testing sets, the dataset was consistently divided into these partitions with an 80:20 ratio. This meant that no data sample present in the training set was ever included in the test set, ensuring an unbiased evaluation of the model performance on the unseen data. Given the unique characteristics of EEG signals and individual subject variability, three distinct data-splitting strategies were employed and evaluated:

Pooled Random Split: This represents the most straightforward and standard data-splitting approach (Figure 1). All individual EEG epochs, collected from all selected subjects, were first pooled into a single, unified dataset. Subsequently, this combined dataset was randomly partitioned into training and testing sets according to the 80:20 ratio. The underlying assumption of this strategy was that each epoch would constitute an independent data sample.

**Figure 2:**
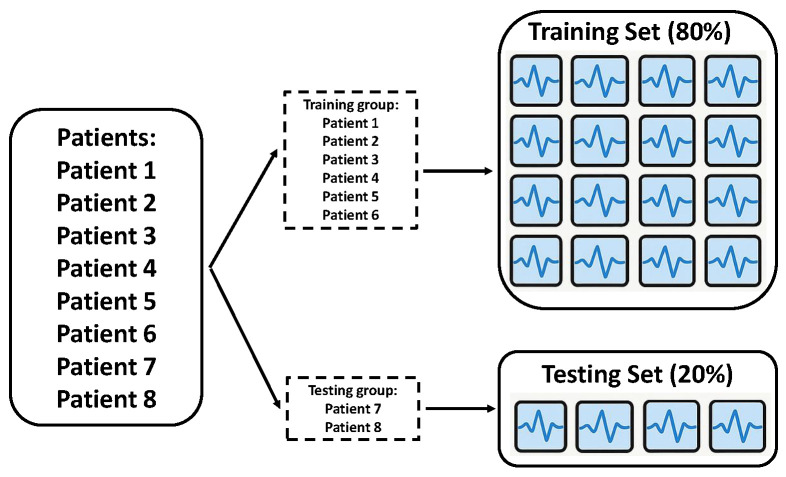
Illustration of Patient Leave-P-Out data-splitting methodology.

Patient Leave-P-Out: This strategy ensured complete patient separation between the training and testing sets, directly addressing the inherent inter-subject variability in EEG data and eliminating any possibility of patient-specific data leakage (Figure 2). Instead of splitting individual epochs, the list of patient identifiers was divided into training and testing groups. All data originating from patients whose IDs were assigned to the training group were exclusively used for training, while all data from patients in the testing ID group were exclusively allocated to the testing set. This method provides a more realistic assessment of a model’s generalization capability to unseen individuals, which is crucial for practical EEG-based BCI applications where a system trained on a specific group of users needs to perform effectively on new, untrained users.

**Figure 3:**
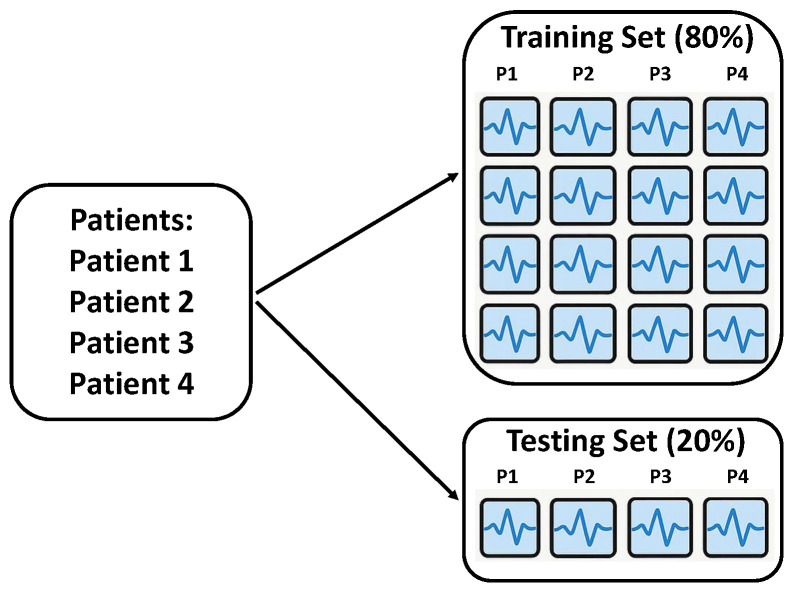
Illustration of Intra-Patient Then Pool data-splitting methodology.

Intra-Patient Then Pool: This hybrid strategy struck a compromise between the two aforementioned approaches (Figure 3). In the initial step, the data of each individual patient were independently divided into a patient-specific training set and a patient-specific testing set, maintaining the 80:20 ratio for each patient. Crucially, this patient-specific split ensured that no epoch from a patient’s training data would appear in their corresponding test data. Subsequently, all patient-specific training sets were concatenated to form the final global training set, and, similarly, all patient-specific testing sets were combined to create the final global testing set. This approach aims to balance the need for subject-specific training while still enabling the model to learn from a diverse pool of patients for generalization.

#### 2.2.2. Data-Augmentation Methods

A comprehensive set of 12 available and popular data-augmentation methods, as described in the scientific literature [38], was employed in this study. These methods are broadly categorized into transformative and generative techniques. Transformative methods, which modify existing EEG epochs, can be further subdivided based on the domain of transformation: temporal-, spatial-, and frequency-based methods. The following sections detail the specific techniques used.

#### 2.2.3. Transformative Augmentation Methods

The transformative methods operate by directly modifying existing EEG epochs to create new ones.

##### Temporal Augmentation (Figure 4)

1. Random Masking: This technique randomly masks (sets to zero) a continuous segment of the signal within a given epoch. A new sample is created with a probability of p=0.5 to ensure a balance between original and augmented data. The length of the masked segment is 10% of the total signal length, a value chosen to simulate short-term artifacts without significantly distorting the underlying neural patterns [51].
2. Time Shifting: This method performs a random, cyclic shift of the entire signal along the time axis. The shift amount is an integer number of samples, randomly chosen from the range [−20,20]. This range, which covers a time span of 0.25 s, was selected to account for minor temporal misalignment of neural activity relative to event markers, a common issue in EEG data.
3. Amplitude Scaling: This method randomly scales the amplitude of an entire EEG epoch. A scaling factor is chosen from a uniform distribution within the range (0.9,1.1). This narrow range was chosen to simulate realistic variations in signal amplitude due to factors such as changes in electrode contact or impedance, without introducing extreme, non-biological signal levels [52].
4. Noise: This technique adds Gaussian Noise to the EEG signal. The standard deviation of the noise is proportional to the standard deviation of the original signal, controlled by a factor of 0.05. This value was chosen to introduce a small amount of noise, improving model robustness against minor electrical interference, without overwhelming the true signal [53].
5. Sign Flip: This method randomly inverts the polarity of the entire EEG signal for an epoch with a probability of p=0.3. This addresses the ambiguity of EEG signal polarity and encourages the model to learn features that are invariant to signal direction [54].

##### Spatial Augmentation (Figure 5)

1. Channels Dropout: This technique randomly sets the values of selected channels to zero for an entire epoch. It is applied with a probability of p=0.5, and the number of channels to drop is 10% of the total channels. This encourages the model to learn features that are distributed across multiple channels, mitigating over-reliance on a few electrodes that may be noisy or faulty [55].
2. Channel Shuffle: For a given epoch, this method randomly shuffles the order of the channels with a probability of p=0.5. This is particularly useful for models with spatial-sensitive layers, as it forces the network to generalize beyond the fixed physical arrangement of electrodes [56].

##### Frequency- and Wavelet-Domain Augmentation (Figure 6)

1. Band-Stop Filter: This technique applies a random Band-Stop Filter to a selected portion of the data with a probability of p=0.5. The filter’s center frequency is chosen from the range [10.0,40.0] Hz, with a bandwidth from [1.0,5.0] Hz, and a filter order of 4. These parameters were chosen to simulate localized frequency artifacts (e.g., muscle activity, eye blinks) within the range of interest for BCI signals [51]. A notch-type digital filter was applied as an augmentation, with the Band-Stop defined by a randomly selected center frequency and bandwidth. The filter was designed using a 4th-order Butterworth prototype and applied with zero-phase forward–backward filtering.
2. DWT-based Augmentation (DWTaug): This method operates in the wavelet domain. A Discrete Wavelet Transform (DWT) is applied using the db4 wavelet, with the decomposition level set to the maximum possible. Gaussian Noise, with a standard deviation scaled by a factor of 0.05, is added to the wavelet coefficients before the signal is reconstructed. This approach effectively introduces realistic, frequency-specific perturbations to the signal without altering its fundamental structure [42].
3. Fourier Surrogate: This augmentation method generates a surrogate signal by performing a Fast Fourier Transform (FFT), randomizing the phase component while keeping the amplitude constant, and then applying an inverse FFT to reconstruct the time-domain signal [57].

#### 2.2.4. Generative Augmentation Methods

Generative methods produce entirely new synthetic signals that mimic the distribution of the original EEG signal.

1. Variational Autoencoder (VAE): This method utilizes a Conditional VAE to generate new EEG epochs. The model is trained for 50 epochs with a batch size of 32 and a learning rate of 0.0002. The latent space dimension is set to 100, a value chosen to balance information compression with the ability to represent the data variability. New epochs are generated by sampling from the latent space and conditioning the decoder on a desired class label [38,58].
2. Generative Adversarial Network (GAN): This method employs a Conditional GAN. The network is trained using the same hyperparameters as the VAE, with a latent noise vector dimension of 100. New samples are generated by feeding random noise and a desired class label to the trained generator. The parameters are aligned with the VAE for a direct comparison of generative model performance [59].

### 2.3. Neural Network Architecture: EEGNet

For the classification task, the convolutional neural network EEGNet was selected as the core model. EEGNet is a well-established and widely used benchmark architecture in the scientific community for EEG signal analysis, designed to efficiently extract both spatial and temporal features from raw EEG data. It is particularly well-suited for tasks involving motor imagery (MI) and motor execution (ME), which were the focus of our study. Based on the original architecture proposed by [50] a custom implementation was designed and built specifically for this research using the PyTorch [60] framework, in contrast to the original’s use of TensorFlow. This implementation leverages EEGNet’s lightweight design, which makes it highly effective for a broad range of EEG classification problems.

**Figure 7:**
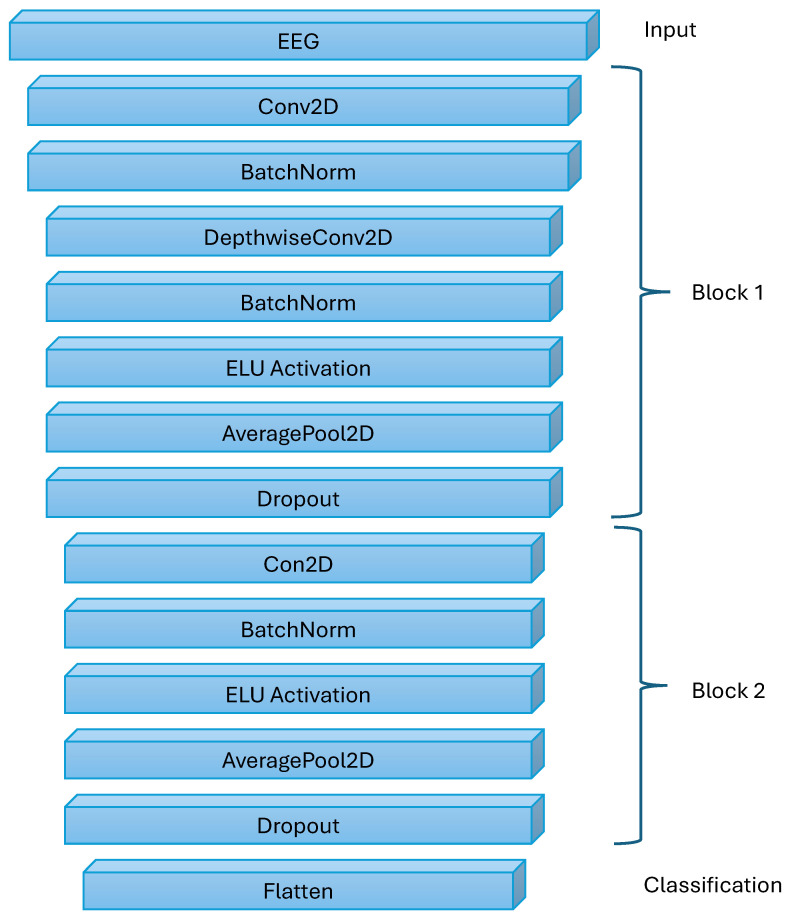
General structure of EEGNet.

All our experiments were conducted using the same EEGNet architecture and training configuration to ensure full reproducibility and comparability of results. The implemented EEGNet model was designed for EEG epochs consisting of 64 channels and 640 temporal samples. The network comprises two convolutional blocks followed by a fully connected classification layer. In the first block, temporal feature extraction is performed using 8 convolutional filters with a kernel size of 1 × 64, followed by batch normalization. Spatial filtering across all EEG channels is achieved using a depthwise convolution with a depth multiplier of 2, resulting in 16 feature maps. This block employs an ELU activation function, average pooling with a kernel size of 1 × 4, and dropout with a rate of 0.5. The second block applies a convolution with 16 filters and a kernel size of 1 × 16, followed by batch normalization, ELU activation, average pooling with a kernel size of 1 × 8, and dropout with a rate of 0.5. After both pooling operations, the temporal dimension is reduced to 20 samples, producing a flattened feature vector of length 320. This vector is passed to a fully connected layer that outputs class scores. The model was trained using the Adam optimizer with a learning rate of 0.001. A categorical cross-entropy loss function was used, and training was performed with a batch size of 32. Early stopping based on training loss was applied with a patience limit of 50 epochs, and the model weights corresponding to the lowest loss were restored. The same network architecture and hyperparameter settings were used across all the experiments. The structure of EEGNet is presented in Figure 7. The values of the EEGNet hyperparameters are summarized in Table 1.

The EEG signals were segmented into fixed-length epochs prior to network input. Epoching was performed using task-related event markers provided in the dataset annotations. For all motor execution and motor imagery classes, epochs were extracted using an identical time window of 640 samples (corresponding to approximately 0.29 s at the acquisition sampling rate), aligned relative to the onset of the corresponding task cue. No overlapping windows were used, and a single epoch was extracted per trial.

Rest-class epochs were constructed using labeled resting intervals temporally separated from task execution periods and matched in duration to task-related epochs. The same window length and preprocessing pipeline were applied consistently across all classes. No trial rejection or artifact-based epoch exclusion was performed at the epoching stage. All extracted epochs were subsequently used as direct inputs to the EEGNet model without further temporal segmentation.

### 2.4. Computational Environment

The entire analytical pipeline, encompassing data loading, preprocessing, deep learning model training, and performance evaluation, was developed using an authorial program written in Python 3.13.4. The implementation was realized with the aid of key scientific computing libraries, including PyTorch 2.7.1 [60] for the neural network framework, alongside such as NumPy, SciPy or Pandas. All computations were executed on two separate machines, both operating under the Windows 11 operating system. The specifications for these machines are as follows:

- Machine 1: ASUS ROG Strix SCAR 17 R9-7945HX + RTX4090
- Machine 2: PC R7-7800X3D + RTX 5070Ti

## 3. Results

All the results presented in this section were derived from a series of rigorous experiments conducted using our program. It executed a total of approximately 15,000 experiments, each representing a unique combination of the data-splitting methods, augmentation techniques, and augmentation strategies detailed in the Materials and Methods section. The experiments were performed using data from the 25 randomly selected patients: S057, S104, S004, S037, S023, S077, S049, S089, S025, S084, S044, S014, S015, S054, S105, S029, S018, S038, S042, S033, S065, S022, S028, S048, and S051. As described in the introduction, the focus of these experiments was the three-class classification problem using motor imagery (MI) data.

To guarantee the statistical robustness of our findings, each tested configuration was repeated 50 times. Training proceeded for a maximum of 500 epochs, with early stopping triggered if no statistically significant improvement appeared after 100 consecutive epochs. Hyperparameters corresponding to the top-performing models were archived in case of future replication or verification.

**Figure 8:**
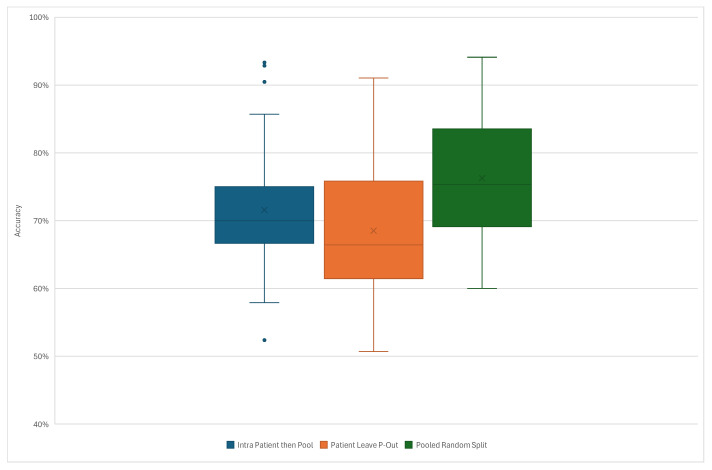
Summary plots showing classification accuracies across three tested data-splitting strategies.

The classification accuracies were calculated for raw EEG data for the three data-splitting strategies in order to define the reference values. The box plots representing the accuracy values for these three strategies are presented in Figure 8.

One-way ANOVA analyses were performed to evaluate the effect of the data-splitting strategies and augmentation methods on classification accuracy. To account for multiple comparisons, all *p*-values within each family of tests were adjusted using the Benjamini–Hochberg false discovery rate (FDR) procedure with α=0.05. Families were defined as pairwise comparisons between split methodologies for each original-to-augmented data ratio. Table 2 presents the raw *p*-values, FDR-adjusted *p*-values and Cohen’s *d* effect sizes for the split strategy comparisons. For the raw EEG data, all the splitting strategies and augmentation ratios exhibited statistically significant differences after FDR correction, while for the preprocessed EEG data, significant differences were observed between specific pairs (Patient Leave-P-Out vs Pooled Random Split, Intra-Patient vs Pooled Random Split for 1:1 ratio). Large Cohen’s *d* values confirmed that these differences were practically meaningful.

### 3.1. Single-Method Augmentation

**Figure 9:**
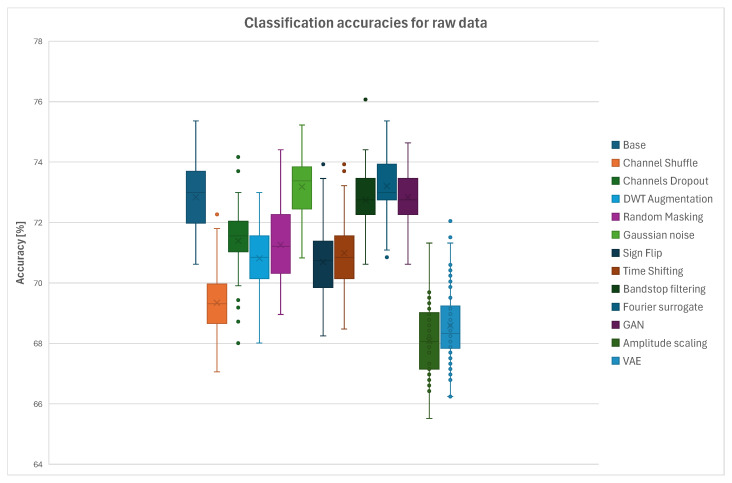
Summary plot of accuracies across all tested augmentation methods for Intra-Patient Then Pool data-splitting scenario using raw EEG signals.

**Figure 10:**
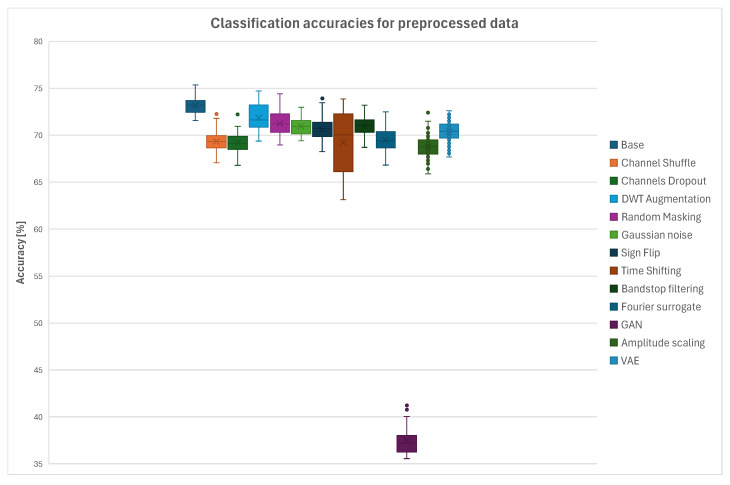
Summary plot of accuracies across all tested augmentation methods for Intra-Patient Then Pool data-splitting scenario using initially notch-filtered EEG signals.

In the first step the augmentation methods were tested separately to verify the stability of the used neural network and subjected to statistical analysis using one-way ANOVA in order to examine the influence of each method on the classification accuracy with respect to the accuracy obtained for the original dataset. Box plots presenting accuracy values for all the augmentation methods for raw data and EEG signals filtered using notch filter are presented in Figure 9 and Figure 10. It can be observed that in case of the raw EEG data used the significant decrease of accuracy was a result of using Channel Shuffle, Amplitude Scaling and VAE algorithms for data augmentation. Minor performance degradation was found for the Channels Dropout, DWTaug, Random Masking, Sign Flip, and Time Shifting methods. Accuracies obtained at the level of the original dataset or slightly better were obtained for the Gaussian Noise, Band-Stop Filtering, Fourier Surrogate and GAN augmentation algorithms. In the case of EEG data filtered using notch filter before uploading them to EEGNet the accuracy values obtained for the tested augmentation methods were significantly different. A significant decrease in accuracy, by almost 40%, was observed for the GAN method, which suggests that the neural network used in this algorithm might be unstable. A mild decrease in accuracy was found in the cases of the Channel Shuffle, Channels Dropout, Fourier Surrogate and Amplitude Scaling methods. The remaining algorithms did not significantly influence the classification accuracies.

The ANOVA analysis was performed in order to verify which methods, compared to the original dataset, showed statistically significant influence on the classification accuracies. Similarly, as in the cases of the data-splitting methodologies analysis, all the *p*-values within each family of tests were adjusted using the Benjamini–Hochberg false discovery rate (FDR) procedure with α=0.05. Families were defined as comparisons of each augmentation method against the original dataset, separately for raw and preprocessed EEG data. Table 3 summarizes the raw *p*-values, FDR-adjusted *p*-values and Cohen’s *d* for each augmentation method compared to the original dataset. For the raw EEG data, the methods Channel Shuffle, Channels Dropout, DWT-based Augmentation, Random Masking, Amplitude Scaling, Sign Flip, Time Shifting, and VAE showed strong effects. For the preprocessed EEG data, all the augmentation methods exhibited substantial effect sizes. These results indicate robust and meaningful improvements, while FDR correction ensured control over false positives due to multiple testing.

The average classification accuracies, median values and corresponding interquartile range (IQR) values for a 1:1 ratio of original to augmented data and raw EEG signals as an input for EEGnet for all the tested data-splitting strategies are presented in Table 4. The tables include the baseline values and results for each tested augmentation method. The observed IQR values indicate relatively low dispersion and good consistency of the measured outcomes.

**Figure 11:**
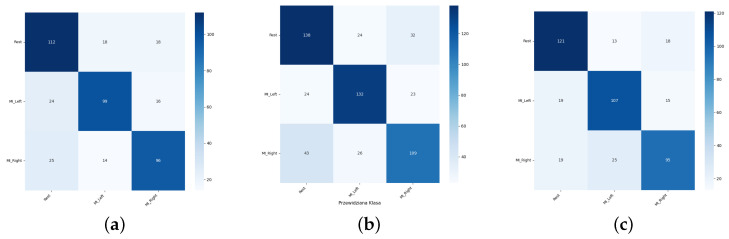
Confusion matrices for the 1:1 DWT augmentation ratio obtained from one randomly selected run among 50 repeated EEGNet training runs: (**a**) Intra-Patient Then Pool, (**b**) Patient Leave-P-Out, and (**c**) Pooled Random Split. Rows correspond to true class labels and columns to predicted labels.

**Figure 12:**
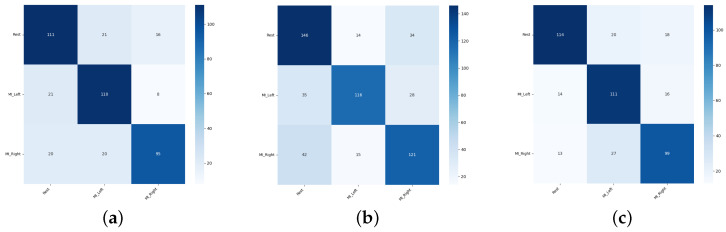
Confusion matrices for the 1:1 Sign Flip augmentation ratio obtained from one randomly selected run among 50 repeated EEGNet training runs: (**a**) Intra-Patient Then Pool, (**b**) Patient Leave-P-Out, and (**c**) Pooled Random Split. Rows correspond to true class labels and columns to predicted labels.

**Figure 13:**
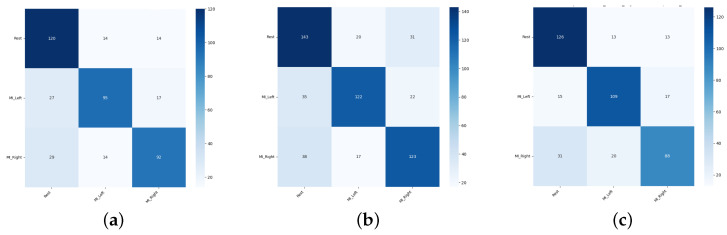
Confusion matrices for the 1:1 Channels Dropout augmentation ratio obtained from one randomly selected run among 50 repeated EEGNet training runs: (**a**) Intra-Patient Then Pool, (**b**) Patient Leave-P-Out, and (**c**) Pooled Random Split. Rows correspond to true class labels and columns to predicted labels.

Sample confusion matrices are presented in Figure 11, Figure 12 and Figure 13 for all three tested data-splitting approaches using a 1:1 augmentation ratio and raw EEG signals. For each splitting strategy the matrices correspond to the best-performing augmentation method from the time-domain, frequency-domain, and spatial-domain categories, illustrating class-wise classification behavior and highlighting differences in the error patterns across the evaluation protocols.

The impact of each augmentation method on classification accuracy was evaluated not only across three data-splitting strategies, but also four original-to-augmented data ratios. At a 1:0.25 ratio, the Intra-Patient Then Pool data-splitting methodology with raw EEG showed no improvement. When using notch-filtered EEG at the input, some improvements were observed with DWT-based Augmentation, Band-Stop Filtering and Random Masking. Under the Patient Leave-P-Out data-splitting approach, the Random Masking and Channel Shuffle augmentation methods improved accuracy by approximately 0.5% for raw EEG, while Fourier Surrogate led to more than 2% improvement of classification accuracy for notch-filtered EEG. The Pooled Random Split method showed no significant gains in accuracy values for raw EEG, and preprocessed EEG exhibited an average decrease of 3.78%. At a 1:0.50 ratio, performance varied across the split methodologies and preprocessing. For the Intra-Patient Then Pool data-splitting approach, all the augmentations decreased classification accuracy by around 6% for raw EEG, whereas the notch-filtered EEG showed larger gains (>2%) with Channels Dropout, DWT-based Augmentation, and Random Masking. The Patient Leave-P-Out data-splitting strategy provided only small accuracy gains (<1.5%) for raw EEG, while notch-filtered EEG benefited modestly from Band-Stop Filtering, Sign Flip, Random Masking, Channel Shuffle, and DWT-based Augmentation.

At a 1:0.75 ratio, the results were more variable. The Intra-Patient Then Pool data-splitting approach with raw EEG led to an average decrease of 5.3% across all the augmentation methods, while notch-filtered EEG showed improvements of around 2% with DWT-based Augmentation and Amplitude Scaling. For the Patient Leave-P-Out data-splitting methodology, accuracy results for raw EEG saw negligible improvement (<0.2%) from Channel Shuffle, whereas most of the other methods decreased accuracy by around 1.1%. Notch-filtered EEG provided modest gains (<1.5%) in accuracy values with Fourier Surrogate and Sign Flip augmentations. Under the Pooled Random Split, raw EEG as an input signal to EEGNet showed only minor benefit (<0.5%) in classification accuracy using Time Shifting augmentation, while for notch-filtered EEG a decrease of approximately 2% in classification accuracy was noted across all the tested augmentation methods.

At a 1:1 ratio of original to augmented data, the overall outcomes were more favorable. The Intra-Patient Then Pool data-splitting approach with a raw EEG signal as the input showed improvements greater than 1% with the Channel Shuffle and Time Flip algorithms. Notch-filtered EEG at the input exhibited around 1% improvement with Channels Dropout and DWT-based Augmentation. For the Patient Leave-P-Out data-splitting approach, the classification of raw EEG signals improved by approximately 1% with the Channels Dropout and Sign Flip augmentation methods, while notch-filtered EEG signals at the input showed similar gains in classification values with Fourier Surrogate and Channels Dropout augmentations. The Pooled Random Split provided minor improvements in classification accuracy, and about 0.5% accuracy gains for notch-filtered EEG.

**Figure 14:**
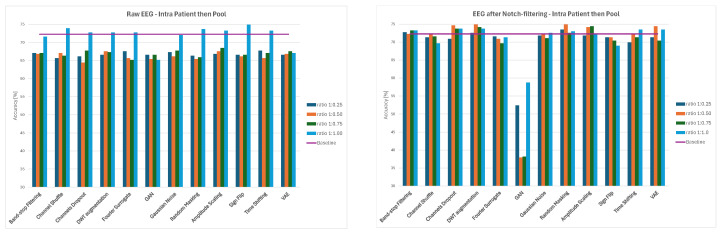
Average classification accuracy values for raw and notch-filtered EEG data in Intra-Patient Then Pool approach across all tested augmentation methods.

**Figure 15:**
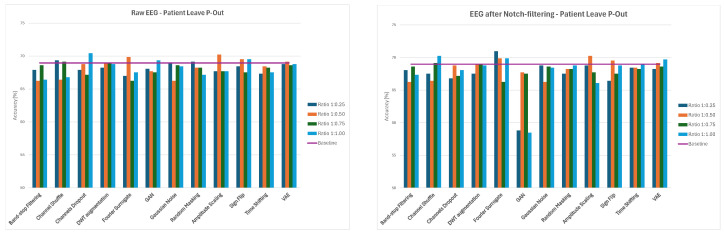
Average classification accuracy values for raw and notch-filtered EEG data in Patient Leave-P-Out approach across all tested augmentation methods.

**Figure 16:**
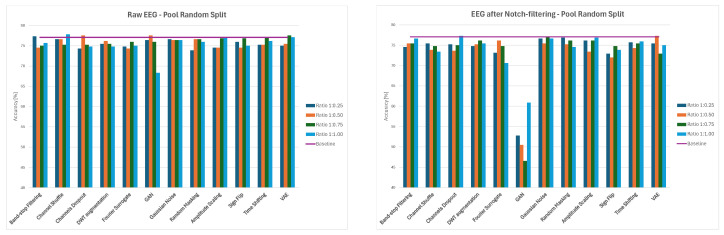
Average classification accuracy values for raw and notch-filtered EEG data in Pooled Random Split approach across all tested augmentation methods.

Overall, the results demonstrate that augmentation effects depend strongly on both preprocessing and data-splitting methodology. While some methods, such as Channels Dropout and DWT-based Augmentation, frequently improved performance—especially at lower augmentation ratios and for notch-filtered EEG—others, including Sign Flip, GAN, or Time Shifting, had mixed or even negative effects depending on the specific combination of preprocessing method and data-splitting strategy. The GAN method consistently led to significant decreases in classification accuracy, ranging from 21–34% for 1:0.50 and 1:0.75 ratios and to around 12% for the 1:1 ratio across all the splitting methods. The average values of classification accuracy for all four ratios of original to augmented data and the three examined strategies are presented in Figure 14, Figure 15 and Figure 16.

### 3.2. Two-Stage Cascade Augmentation

In the next stage of classification results analysis the combinations of two different augmentation methods were considered. The analysis was divided with respect to the data-splitting strategies, considering raw EEG signals as the input to the EEGNet and EEG data filtered using notch filter.

#### 3.2.1. Two-Stage Intra-Patient Then Pool

The results obtained for the raw EEG signal as the input to the EEGNet for the two-stage augmentation and Intra-Patient Then Pool data-splitting strategy show that data augmentation reliably improved classification performance over raw EEG for three-class motor imagery when using EEGNet, but that the choice of augmentation method was very important. The highest-performing combinations consistently involved spectral and spatial augmentations, such as Fourier Surrogate, DWT augmentation, Channels Dropout, and Channel Shuffle.

In contrast, simple combinations such as Gaussian Noise addition, Amplitude Scaling, or Sign Flip tended to produce only modest gains in classification accuracy, and the generative models alone did not outperform the traditional augmentation methods. Overall, the strongest and most consistent improvements were obtained with spatial + spectral augmentation pairs, followed by spatial + temporal and spectral + temporal combinations. As the ratio of original-to-augmented data increased from 1:0.25 to 1:1, the variability ranking remained stable. Consistently strongest was the combination of DWT-based Augmentation with Time Shifting (increasing in accuracy by around 2%). What should be avoided is combining two augmentation methods that are noise-based and combinations of two generative methods.

**Figure 17:**
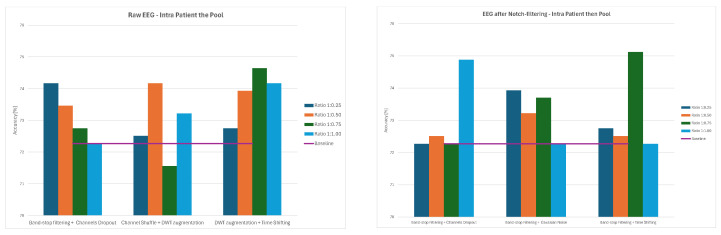
Average classification accuracy values for raw and notch-filtered EEG data in Intra-Patient Then Pool approach for best three two-stage cascades.

Applying a notch filter to remove power line noise produced clear and consistent improvement in classification accuracy for Intra-Patient Then Pool data-splitting. Notch-filtered data reached roughly 74–76%. The largest benefits were observed for spectral–spatial combinations, which frequently exceeded 75% accuracy after filtering. Spectral–temporal and spatial–heavy augmentations also showed consistent though slightly smaller gains. Generative approaches such as GANs or VAEs became more stable with notch filtering. This implies that notch filtering should be a standard preprocessing step, paired with structured spectral, spatial, and temporal augmentations. The best three two-stage cascade combinations of augmented methods are presented in Figure 17.

#### 3.2.2. Two-Stage Patient Leave-P-Out

Under the Patient Leave-P-Out data-splitting strategy, the baseline classification accuracy was approximately 69%. Data augmentation for the raw EEG signal as the input to EEGNet provided only modest improvements, with most of the augmented models achieving accuracies between 65.5% and 70.5%. The potential for improvement was very limited, with gains of only 0–2%. This behavior was expected, as cross-subject generalization is highly challenging, and aggressive augmentation strategies often degrade model performance rather than improve it. The most effective approaches combined spectral and spatial augmentations, which consistently achieved the highest performance (around 70–71%). These methods worked because they introduced realistic inter-subject variability in both the frequency characteristics and the spatial patterns. Combinations of spectral and temporal augmentations also performed well, frequently matching or slightly exceeding the baseline. Spatial and temporal combinations provided more moderate but reliable gains, typically reaching near-baseline performance. Noise-based transformations and simple or redundant augmentations often reduced accuracy well below the baseline, as they introduced unstructured distortions that did not reflect real inter-subject differences. Similarly, generative models used alone or combined with noise or scaling tended to produce unrealistic samples and led to poorer generalization. Several strategies should be avoided.

**Figure 18:**
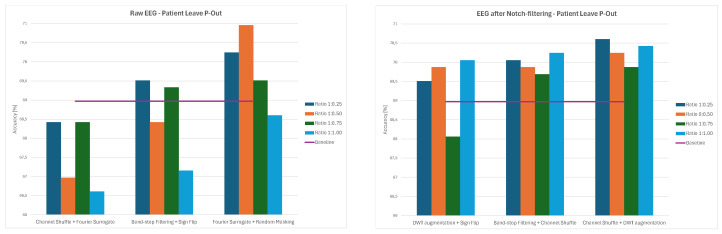
Average classification accuracy values for raw and notch-filtered EEG data in Patient Leave-P-Out approach for best three two-stage cascades.

Applying a notch filter in the leave-p-out setting led to a clear but modest improvement in performance. The distribution of results became smoother, with fewer models falling below the baseline. The largest benefits from notch filtering occurred in the spectral-dependent augmentations, where noise removal stabilized the frequency-domain transformations and reduced performance collapsed. Overall, notch filtering reduced catastrophic failures, improved median accuracy by about 0.8% across the augmentations (and by up to 1.2% for the spectral methods), and strengthened the same augmentation families that were already effective. Consequently, notch filtering is recommended as a stabilizing preprocessing step for Patient Leave-P-Out data splitting, paired with structured spectral, spatial, and temporal augmentations, while generative and noise-heavy strategies should still be avoided. The best three two-stage cascade combinations of augmented methods are presented in Figure 18.

#### 3.2.3. Two-Stage Pooled Random Split

With the Pooled Random Split strategy and a raw EEG signal, data augmentation led to moderate but consistent improvements over the baseline. Most augmentations were beneficial or at least harmless, typically improving accuracy by about 1–2.5%. The strongest augmentation families consistently combined structured transformations. Spectral and spatial augmentations were the most reliable and frequent top performers. Spectral and temporal combinations also performed strongly. Spatial and temporal pairs provided stable, near-top performance. The generative models contributed positively only when paired with meaningful structured augmentations. Poor results were mainly associated with GAN-only or weakly constrained GAN combinations, as well as noise-based or spatial-only augmentations, which introduced unstructured variability and significantly degraded performance.

**Figure 19:**
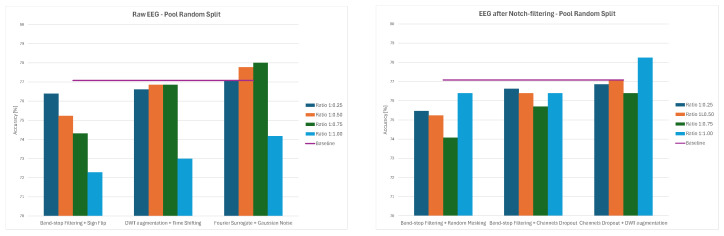
Average classification accuracy values for raw and notch-filtered EEG data in Pooled Random Split approach for best three two-stage cascades.

Notch filtering had a positive impact on pooled-random generalization, making it the evaluation setting that benefited the most from noise removal. Catastrophic failures largely disappeared, except for the GAN-based pipelines, which remained unstable. The best three two-stage cascade combination of augmented methods are presented in Figure 19.

#### 3.2.4. Two-Stage Augmentation Summary

**Figure 20:**
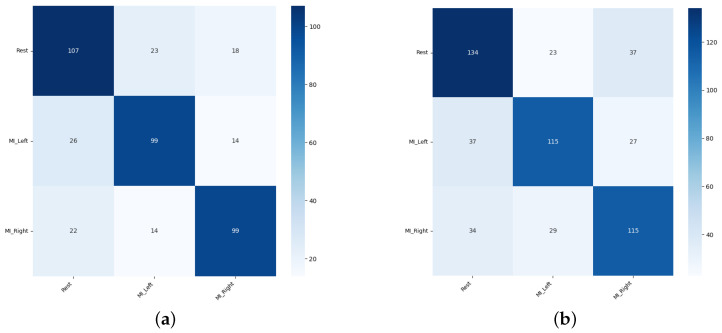
Confusion matrices for the 1:1 augmentation ratio obtained from one randomly selected run among 50 repeated EEGNet training runs: (**a**) Intra-Patient Then Pool split, Band-Stop Filtering + Channels Dropout; (**b**) Patient Leave-P-Out split, Channel Shuffle + DWT augmentation. Rows correspond to true class labels and columns to predicted labels.

Table 5 presents a list of the five best two-stage augmentation combinations for raw and notch-filtered EEG data and all three data-splitting strategies: Intra-Patient Then Pool (IPP), Patient Leave-P-Out (PLO) and Pooled Random Split (PRS). Our key observations indicate that notch filtering substantially boosted pooled-random performance. The classification accuracies for raw EEG peaked around 73–74%, while the notch-filtered data led to achieving classification accuracy of around 77–78%, representing absolute gains of 3–4% for all the top-performing augmentations. In Figure 20 the example confusion matrices are presented for representative two-stage cascades for the Intra-Patient Then Pool and for the Patient Leave-P-Out splitting strategies.

Overall, these findings support two practical conclusions. First, notch filtering should be treated as a mandatory preprocessing step in MI–EEG pipelines, especially when employing spectral augmentations or training with pooled multi-subject data. Second, the combination of notch filtering with physically meaningful augmentations (e.g., Band-Stop Filter + DWT augmentation, Channels Dropout + Time Shifting, Gaussian Noise + Amplitude Scaling) yields the best performance for the Pooled Random Split strategy, approaching accuracies near 78%. These results reinforce the value of principled signal cleaning and targeted augmentation design in improving EEG-based motor-imagery classification.

### 3.3. Three-Stage Cascade Augmentation

Similarly, as for two-stage cascade augmentation, an analysis of classification accuracy was performed for three-stage cascades.

#### 3.3.1. Three-Stage Intra-Patient Then Pool

**Figure 21:**
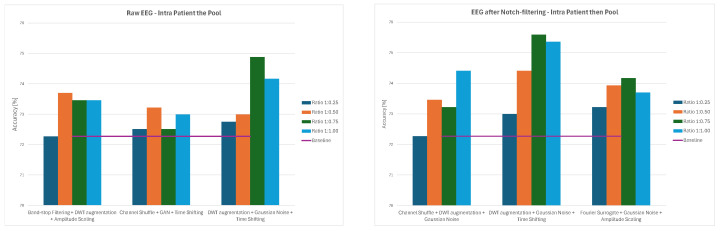
Average classification accuracy values for raw and notch-filtered EEG data in Intra-Patient Then Pool approach for the three best three-stage cascades.

Across three-stage EEG augmentation for the Intra-Patient Then Pool data-splitting strategy, raw and notch-filtered settings exhibited contrasting but complementary behaviors. For raw EEG, the classification accuracy improved monotonically with the augmentation ratio, peaking at the full 1.0:1 ratio. In this setting, spectral augmentations, especially Fourier Surrogate, DWT-based methods and Band-Stop Filtering, consistently dominated the top-performing pipelines, reflecting the frequency-driven nature of motor-imagery EEG. Spatial augmentations acted as early-stage regularizers, while the amplitude, temporal, and noise-based methods provided secondary gains. In contrast, for notch-filtered EEG, multi-domain combinations dominated, with the temporal augmentation methods (Time Shifting, Amplitude Scaling, Sign Flip, Random Masking) and spatial/channel-level augmentations appearing universally. As the ratio increased, the spectral methods regained importance, complementing the temporal and spatial methods.The three best three-stage cascade combinations of augmented methods are presented in Figure 21.

#### 3.3.2. Three-Stage Patient Leave-P-Out

**Figure 22:**
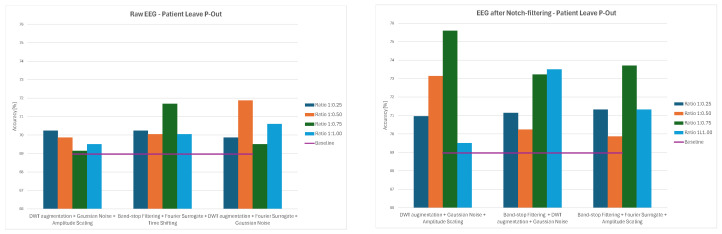
Average classification accuracy values for raw and notch-filtered EEG data in Patient Leave-P-Out approach for the three best three-stage cascades.

Considering three-stage cascade augmentation for the Patient Leave-P-Out data-splitting approach, the raw and notch-filtered EEG exhibited similar performance but differed in stability and augmentation sensitivity. For the raw EEG, classification accuracy improved gradually with the increasing augmentation ratio. In this setting, Fourier- and DWT-based Spectral Augmentations acted as universal backbone methods across all the ratios, while noise addition, masking, and channel-level augmentations became increasingly beneficial as data volume grew. The generative methods remained weak and non-essential. For the notch-filtered EEG, performance was driven more by augmentation composition rather than by ratio. The best combination appeared to be DWT-based Augmentation combined with Fourier Surrogate and Band-Stop Filtering, followed by the Amplitude Scaling and temporal augmentation methods. Across both preprocessing conditions, the most reliable pipelines consistently followed a tri-modal structure combining frequency-domain transformation, filtering, and stochastic amplitude or temporal variation. The three best three-stage cascade combinations of augmented methods are presented in Figure 22.

#### 3.3.3. Three-Stage Pooled Random Split

**Figure 23:**
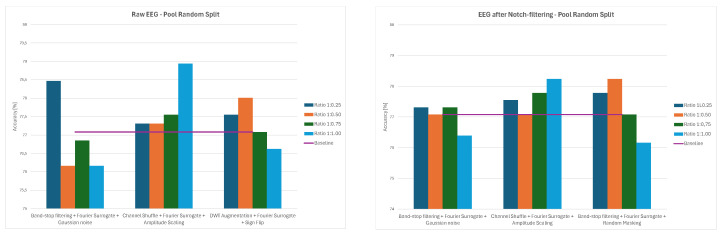
Average classification accuracy values for raw and notch-filtered EEG data in Pooled Random Split approach for the three best three-stage cascades.

In the three-stage Pooled Random Split approach, the raw and notch-filtered EEG again showed contrasting sensitivities to augmentation ratios while sharing a common optimal augmentation structure. For the raw EEG a stable backbone of classical spectral and stochastic augmentations, including Band-Stop Filtering, DWT-based Augmentation, Fourier Surrogate, Gaussian Noise addition, Random Masking, Amplitude Scaling, and Time Shifting, consistently drove strong performance. GAN and VAE were highly ratio-sensitive and often degraded the performance of the classifier. For the notch-filtered EEG, performance became largely insensitive to the augmentation ratio. Here, the best pipelines converged toward deterministic, multi-domain combinations spanning frequency-domain surrogates, filtering, noise addition or temporal augmentations, and channel-level regularization. The strongest pooled-random pipelines followed a consistent three-part structure–spectral transformation + noise/masking + temporal or spatial modification. The three best three-stage cascade combinations of augmented methods are presented in Figure 23.

#### 3.3.4. Three-Stage Cascade Augmentation Summary

**Figure 24:**
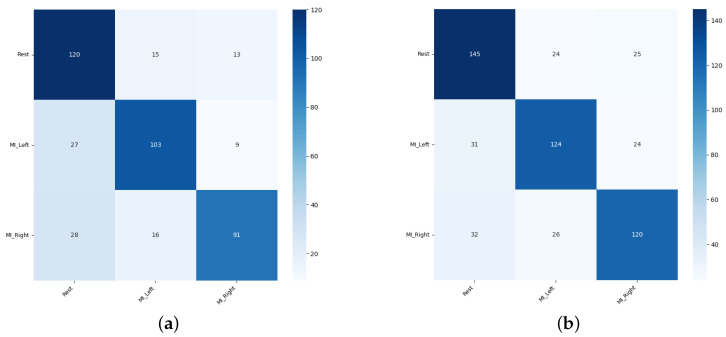
Confusion matrices for the 1:1 augmentation ratio obtained from one randomly selected run among 50 repeated EEGNet training runs: (**a**) Intra-Patient Then Pool split, Channel Shuffle + Fourier Surrogate + Random Masking; (**b**) Patient Leave-P-Out split, Channel Shuffle + DWT augmentation + Sign Flip. Rows correspond to true class labels and columns to predicted labels.

Across all the experiments, EEG classification performance was driven primarily by augmentation choice and structure, not by augmentation ratio, with consistent patterns observed for both raw and notch-filtered EEG across the Patient Leave-P-Out, Intra-Patient Then Pool, and Pooled Random data splits. The frequency-domain augmentations (DWT-based Augmentation and Fourier Surrogate) were universally the strongest contributors, further stabilized by Band-Stop Filtering, Gaussian Noise addition and Random Masking, and light temporal or signal amplitude manipulations. Notch filtering systematically improved stability and top-end accuracy. The channel-level augmentations (Channel Shuffle and Channels Dropout) were most beneficial when subject mixing was high; they were moderately useful in case of Intra-Patient Then Pool data-splitting strategy; and they were least consistent in the Patient Leave-P-Out approach. The GAN and VAE augmentations were non-essential. Overall, the optimal EEG augmentation strategy across all the settings was a multi-domain triplet combining spectral surrogate + filtering + noise/temporal variation. Two sample confusion matrices representative of three-stage augmentation in Intra-Patient Then Pool and Patient Leave-P-Out data-splitting approaches are presented in Figure 24.

The top five three-stage EEG augmentation combinations across the analyzed data-splitting strategies are presented in Table 6. Taking into consideration cross-strategy robustness, recurrence in top results and peak classification accuracy stability, a set of five universal three-stage augmentation cascades were selected and are summarized in Table 7. Note that all the universally best three-stage cascades follow the same structure: spectral surrogate, filtering or noise addition, amplitude or spatial refinement.

## 4. Discussion

**Figure 25:**
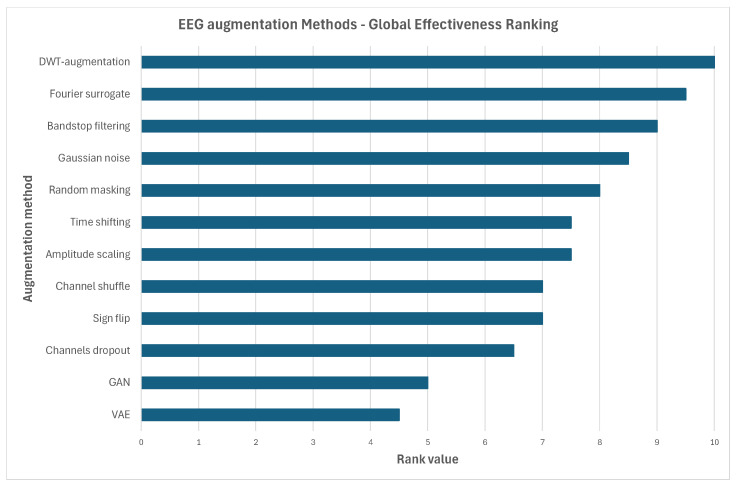
Relative effectiveness of all tested data-augmentation algorithms.

Our results demonstrate that the effectiveness of EEG data augmentation is highly dependent on the interaction between preprocessing, augmentation composition, the data-splitting strategy, and the original-to-augmented data ratio, rather than on any single factor in isolation. Across both two-stage and three-stage augmentation settings, structured, physiologically grounded transformations—particularly spectral (Fourier Surrogate, DWT-based Augmentation), spatial (Channel Shuffle and Channels Dropout), and carefully controlled time-domain methods—consistently outperformed simpler amplitude-based or noise-only methods. In the Intra-Patient Then Pool data-splitting approach, augmentation was most effective when it reshaped subject-specific feature distributions, with the raw EEG benefiting from higher augmentation ratios and spectrally driven pipelines, while the notch-filtered EEG achieved stable peak performance across ratios through richer multi-domain augmentation. In contrast, when selecting data according to the Patient Leave-P-Out approach, our experiments revealed the intrinsic difficulty of cross-subject generalization: the improvements were modest, the gains were limited to specific augmentation families, and aggressive or poorly constrained augmentations often degraded performance. Here, deterministic frequency-domain methods acted as a universal backbone, with noise addition, masking, and channel-level manipulations becoming beneficial only as the data volume increased. The Pooled Random Split approach represented the most augmentation-tolerant scenario, where structured augmentations yielded consistent classification accuracy gains and where notch filtering showed the highest overall performance by stabilizing the spectral and temporal transformations. Across all the strategies, the generative models such as GANs and VAEs were unreliable when used alone and were frequently harmful, particularly at moderate-to-high augmentation ratios, suggesting that they often fail to preserve a physiologically meaningful EEG structure. Notch filtering turned out to be a critical preprocessing step, systematically improving robustness, reducing variance, and amplifying the benefits of the effective augmentation families, especially in the pooled and intra-patient settings. Overall, the findings indicate that optimal EEG augmentation should prioritize deterministic, multi-domain signal-processing transformations, carefully matched to the evaluation protocol, while avoiding excessive or unconstrained augmentation that distorts the underlying neurophysiological distributions. To summarize the findings, a global ranking of the tested augmentation methods was prepared, summarizing augmentation effectiveness across all the experiments. The numeric values between 0 and 10 with step 0.5 were estimated as ordinal proxies, not measured quantities, encoding relative rank spacing and confidence in dominance. The best method achieved the highest rank value, namely 10. The results are presented in Figure 25. Moreover, a heatmap of the analyzed augmentation algorithms was generated, considering the importance of the particular method both as a single augmentation tool and as a part of a cascade. The summary is presented in Table 8.

Our results fit within the prior research on EEG data augmentation, where simple augmentation techniques (e.g., Gaussian Noise addition, sliding windows) have proven more reliable than complex generative methods [44]. For example, GAN-driven strategies such as DCGAN-GP and VAE-based augmentation have shown modest gains (1–5%) in emotion recognition and MI tasks [61] that are consistent with our observation that certain generative approaches offer potential but that their utility is highly context-dependent.

## 5. Conclusions

This study demonstrates that the effectiveness of data augmentation in EEG classification depends strongly on the chosen method, preprocessing, and evaluation protocol. Among single-method augmentations, adding Gaussian Noise, Band-Stop Filtering, DWT-based Augmentation, and Channel Shuffle consistently improved performance in Intra-Patient Then Pool data-splitting scenarios, while Random Masking and Time Shift augmentation were also effective when applied to initially notch-filtered data. Patient Leave-P-Out splitting showed smaller but generally consistent benefits from Channels Dropout, GAN, Gaussian Noise, DWTaug, and Fourier Surrogate augmentation. In the Pooled Random Split scenario, several methods improved raw data accuracy, but many single-method augmentations—notably GAN with notch-filtered input—caused substantial performance reductions, highlighting that single-method gains are conditional on preprocessing and the splitting strategy.

Cascade augmentations produced mixed outcomes. Certain two- and three-stage combinations, such as DWTaug with Gaussian Noise, Channels Dropout with Gaussian Noise, or Gaussian Noise with Random Masking or Amplitude Scaling, yielded accuracy gains above 1.5% in Intra-Patient Then Pool and Pooled Random Split settings. However, many cascades—particularly those involving Sign Flip, Fourier Surrogate, or GAN—often degraded performance, with the Pooled Random Split approach showing the most severe reductions, e.g., Channels Dropout combined with Fourier Surrogate reducing accuracy to 35%.

Considering performance across preprocessing conditions, data-splitting strategies, and augmentation ratios, the most reliable single augmentation methods under the tested conditions were DWT-based Augmentation, Fourier Surrogate, and Channels Dropout. Robust two-stage cascades combined complementary domains, including DWT-based Augmentation with Time Shifting, Fourier Surrogate followed by Channel Shuffle, and DWT-based Augmentation with Channels Dropout. For three-stage cascades, optimal performance was achieved by multi-domain pipelines centered on DWT or Fourier Surrogate, combined with Band-Stop Filtering and either noise addition or Random Masking, optionally enhanced with Channel Shuffle or Channels Dropout, which consistently delivered high classification accuracy and stability across the evaluated scenarios.

These findings indicate that while augmentation can enhance EEG classification, especially with single-method approaches, the benefits are highly dependent on preprocessing, the data-splitting strategy, and the augmentation type. Complex cascade augmentations should be applied cautiously, particularly in Pooled Random Split scenarios, where performance collapses were observed for certain methods.

## Figures and Tables

**Figure 4:**
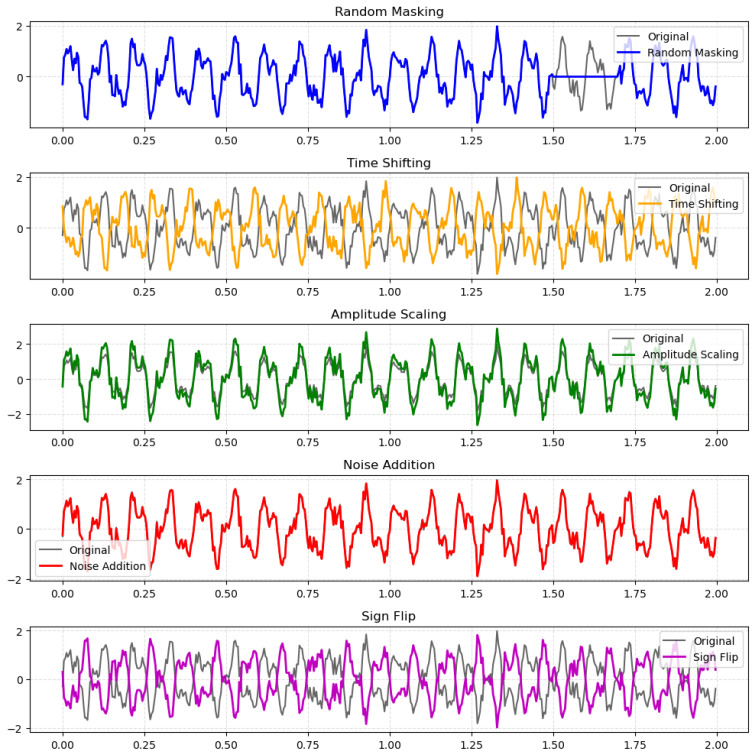
Visualization of the time-domain augmentation methods.

**Figure 5:**
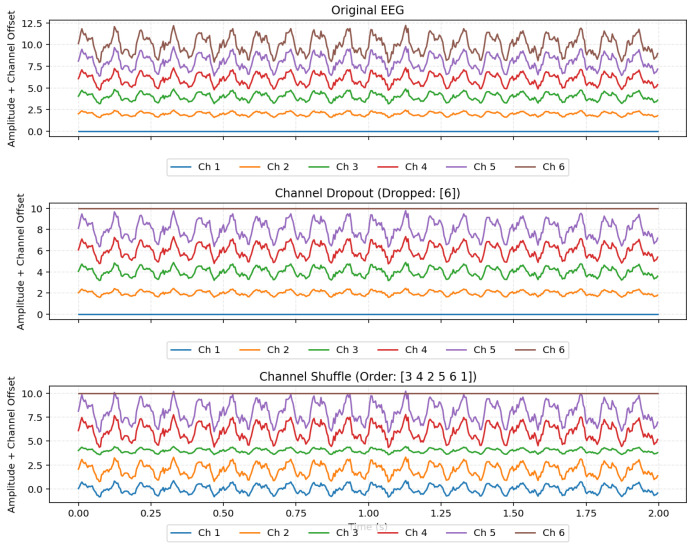
Visualization of the spatial augmentation methods.

**Figure 6:**
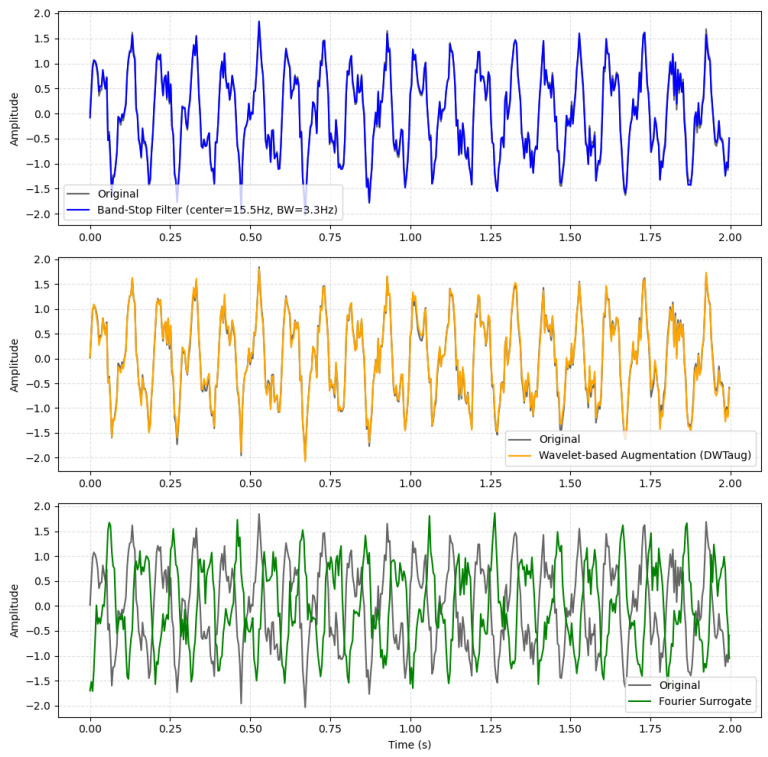
Visualization of the frequency-domain augmentation methods.

**Table 1:** EEGNet architecture and training hyperparameters.

Category	Parameter	Value
Input	Number of channels	64
	Samples per epoch	640
Architecture	Temporal filters ($F_{1}$)	8
	Temporal kernel size	1×64
	Depth multiplier (*D*)	2
	Spatial feature maps	16
	Second block filters ($F_{2}$)	16
	Second block kernel size	1×16
	Activation function	ELU
	Pooling type	Average pooling
	Pooling size (Block 1)	1×4
	Pooling size (Block 2)	1×8
	Dropout rate	0.5
Classifier	Flattened feature size	320
	Output layer	Fully connected
Training	Optimizer	Adam
	Learning rate	0.001
	Batch size	32
	Loss function	Categorical cross-entropy
	Early stopping criterion	Training-loss plateau
	Early stopping patience	50 epochs
	Model selection	Minimum training loss
	Hyperparameters across experiments	Identical

**Table 2:** Results of one-way ANOVA analysis and effect sizes for data-splitting methodologies and different ratios of original-to-augmented data.

Ratio	Split Comparison	Raw EEG *p*-Value (BH-FDR)	Raw EEG Cohen’s *d*	Preprocessed EEG *p*-Value (BH-FDR)	Preprocessed EEG Cohen’s *d*
1:0.25	Intra ^1^ vs. Leave ^2^	$2\times 10^{-5}$ ($4\times 10^{-5}$)	2.308	0.146 (1.000)	0.642
	Leave ^2^ vs. Pooled ^3^	$3\times 10^{-15}$ ($5\times 10^{-15}$)	8.263	0.008 (0.016)	1.229
	Intra vs. Pooled	$2\times 10^{-17}$ ($3\times 10^{-17}$)	10.501	0.212 (0.424)	0.548
1:0.50	Intra vs. Leave	$2\times 10^{-4}$ ($3\times 10^{-4}$)	1.834	0.342 (0.456)	0.414
	Leave vs. Pooled	$2\times 10^{-12}$ ($2\times 10^{-12}$)	6.033	0.049 (0.049)	0.887
	Intra vs. Pooled	$2\times 10^{-16}$ ($3\times 10^{-16}$)	9.303	0.485 (0.485)	0.302
1:0.75	Intra vs. Leave	0.006 ($6\times 10^{-3}$)	1.284	0.337 (0.456)	0.418
	Leave vs. Pooled	$4\times 10^{-17}$ ($1\times 10^{-16}$)	10.124	0.032 (0.043)	0.973
	Intra vs. Pooled	$6\times 10^{-18}$ ($2\times 10^{-17}$)	11.020	0.331 (0.441)	0.423
1:1	Intra vs. Leave	$6\times 10^{-11}$ ($2\times 10^{-10}$)	5.007	0.037 (0.148)	0.946
	Leave vs. Pooled	$2\times 10^{-14}$ ($3\times 10^{-14}$)	7.469	0.001 (0.004)	1.645
	Intra vs. Pooled	$1\times 10^{-7}$ ($1\times 10^{-7}$)	3.240	0.134 (0.424)	0.663

^1^ Intra-Patient Then Pool, ^2^ Patient Leave-P-Out, ^3^ Pooled Random Split.

**Table 3:** Results of the one-way ANOVA for tested augmentation methods compared to original dataset for raw and preprocessed EEG data.

Original Data	Raw EEG *p*-Value (BH-FDR)	Raw EEG Cohen’s *d*	Notch Filter *p*-Value (BH-FDR)	Notch Filter Cohen’s *d*
Band-Stop Filtering	0.686 (0.748)	3.082	$5\times 10^{-19}$ ($6\times 10^{-19}$)	2.246
Channel Shuffle	$8\times 10^{-28}$ ($3\times 10^{-27}$)	3.104	$3\times 10^{-34}$ ($1\times 10^{-33}$)	3.804
Channels Dropout	$2\times 10^{-8}$ ($4\times 10^{-8}$)	1.226	$2\times 10^{-33}$ ($7\times 10^{-33}$)	3.695
DWTaug	$2\times 10^{-15}$ ($5\times 10^{-15}$)	1.915	$9\times 10^{-7}$ ($9\times 10^{-7}$)	1.055
Fourier Surrogate	0.080 (0.107)	0.356	$2\times 10^{-30}$ ($4\times 10^{-30}$)	1.055
GAN	0.961 (0.961)	0.010	$4\times 10^{-117}$ ($5\times 10^{-116}$)	9.941
Gaussian Noise	0.096 (0.115)	0.339	$6\times 10^{-22}$ ($1\times 10^{-21}$)	2.517
Random Masking	$5\times 10^{-9}$ ($8\times 10^{-9}$)	1.299	$2\times 10^{-13}$ ($2\times 10^{-13}$)	1.732
Amplitude Scaling	$3\times 10^{-38}$ ($4\times 10^{-37}$)	4.267	$4\times 10^{-38}$ ($2\times 10^{-37}$)	4.255
Sign Flip	$4\times 10^{-14}$ ($9\times 10^{-14}$)	1.786	$2\times 10^{-19}$ ($4\times 10^{-19}$)	2.274
Time Shifting	$2\times 10^{-11}$ ($3\times 10^{-11}$)	1.542	$3\times 10^{-12}$ ($4\times 10^{-12}$)	1.603
VAE	$1\times 10^{-31}$ ($8\times 10^{-31}$)	3.498	$3\times 10^{-25}$ ($6\times 10^{-25}$)	2.843

**Table 4:** Classification accuracy values and corresponding computation times for single-method augmentation in all data-splitting approaches.

	Intra-Patient Then Pool		Patient Leave-P-Out		Pooled Random Split	
Augmentation	Mean Acc. [%]	Median (IQR)	Mean Acc. [%]	Median (IQR)	Mean Acc. [%]	Median (IQR)
None	71.56	71.52 (1.91)	68.42	68.45 (1.12)	76.16	76.17 (1.43)
Band-Stop Filtering	71.56	71.53 (1.18)	66.42	67.15 (1.68)	75.69	75.34 (1.21)
Channel Shuffle	73.94	72.71 (2.60)	66.79	67.97 (2.49)	77.78	76.73 (0.46)
Channels Dropout	72.75	69.94 (3.32)	70.42	68.33 (1.49)	74.77	75.11 (1.32)
DWTaug	72.75	71.42 (1.72)	68.78	68.87 (0.32)	74.77	75.69 (0.70)
Fourier Surrogate	72.75	70.59 (3.32)	67.51	67.24 (1.31)	75.00	74.88 (0.40)
GAN	72.75	71.59 (1.83)	69.33	67.61 (0.28)	75.46	76.16 (0.86)
Gaussian Noise	72.04	70.53 (1.83)	68.42	68.51 (0.81)	76.39	76.50 (0.28)
Random Masking	73.70	71.11 (2.42)	67.15	68.24 (0.50)	75.93	76.16 (1.04)
Amplitude Scaling	73.22	71.01 (2.30)	67.70	67.7 (0.63)	77.08	74.88 (1.15)
Sign Flip	74.88	72.59 (2.19)	69.51	68.96 (1.31)	75.00	75.46 (1.21)
Time Shifting	72.22	70.41 (2.42)	67.51	67.87 (0.82)	76.16	75.69 (1.27)
VAE	72.51	70.18 (2.02)	68.78	68.69 (1.12)	77.08	76.27 (1.73)

**Table 5:** Top Five Best 2-Stage Augmentation Cascades.

Rank	Combination	Effective Splits	Ratio Sensitivity	Key Reason for Ranking
1	DWTaug + Gaussian noise	All	Very Low	Most stable combination, strong frequency realism, robust regularization.
2	Fourier Surrogate + Gaussian Noise	All	Very Low	Preserves MI structure while injecting realistic variability.
3	Band-Stop Filter + DWTaug	LPO, PRS	Low	Very good frequency shaping, especially after notch filtering.
4	Fourier Surrogate + Amplitude Scaling	IPP, PRS	Low–medium	Strong amplitude robustness without distorting the phase.
5	Channel Shuffle + Gaussian Noise	IPP, PRS	Medium	Effective when subject mixing is high.

**Table 6:** Top five best three-stage augmentation cascades for each data-splitting approach.

Rank	Intra-Patient Then Pool	Patient Leave-P-Out	Pooled Random Split
1	DWTaug + Gaussian Noise + Time Shifting	DWTaug + Gaussian Noise + Amplitude Scaling	Fourier Surrogate + Band-Stop Filtering + Gaussian Noise
2	GAN + Time shift + Channel Shuffle	DWTaug + Fourier Surrogate + Gaussian Noise	Channel Shuffle + Fourier Surrogate + Amplitude Scaling
3	Fourier Surrogate + Gaussian Noise + Amplitude Scaling	Band-Stop Filtering + DWTaug + Gaussian Noise	DWTaug + Fourier Surrogate + Sign Flip
4	Channel Shuffle + DWTaug + Gaussian Noise	Fourier Surrogate + Band-Stop Filtering + Time Shifting	Band-Stop Filtering + Fourier Surrogate + Random Masking
5	Band-Stop Filtering + DWTaug + Amplitude Scaling	Fourier Surrogate + Band-Stop Filtering + Amplitude Scaling	DWTaug + Gaussian Noise + Time Shifting

**Table 7:** Top five universal three-stage augmentation cascades.

Rank	3-Stage Combination	Advantages
1	DWTaug + Band-Stop Filtering + Gaussian Noise	Strongest and most stable across all data-splitting strategies; dominant frequency shaping; robust.
2	Fourier Surrogate + Band-Stop Filtering + Random Masking	Very good generalization; preserves physiological characteristics while improving noise; missing data tolerance.
3	DWTaug + Fourier Surrogate + Gaussian Noise	Very high spectral diversity.
4	Fourier Surrogate + Gaussian Noise + Amplitude Scaling	Simple, deterministic, very stable across original to augmented data ratios.
5	Channel Shuffle + Fourier Surrogate + Amplitude Scaling	Best combination including spatial manipulations.

**Table 8:** Augmentation methods ranking.

Augmentation Method	Intra-Patient Then Pool	Patient Leave-P-Out	Pooled Random Split
DWTaug	Very high	Very high	Very high
Fourier Surrogate	Very high	Very high	Very high
Band-Stop Filtering	High	Very high	Very high
Gaussian Noise	High	High	Very high
Random Masking	High	High	High
Amplitude Scaling	High	High	High
Time Shifting	High	Moderate	High
Sign Flip	Moderate	Moderate	High
Channel Shuffle	High	Moderate	Very high
Channels Dropout	Moderate	Moderate	High
GAN	High	Low	Harmful
VAE	Moderate	Low	Harmful

## Data Availability

Data were obtained from an open-source database available at https://physionet.org/content/eegmmidb/1.0.0/ (accessed on 30 June 2025).
